# Advanced hybrid 3DCNN-SGAN framework for high-precision gas mixture analysis with sensor arrays

**DOI:** 10.1038/s41598-026-41434-1

**Published:** 2026-03-05

**Authors:** Ghazala Ansari, Rupali Singh, Sachin Kumar, Ravi Kumar, U. Siddaraj

**Affiliations:** 1 Department of Electronics and Communication Engineering, Faculty of Engineering and Technology, SRM Institute of Science and Technology, Delhi-NCR Campus, Delhi-Meerut Road, Modinagar, Ghaziabad, Uttar Pradesh 201204 India; 2 Department of Electronics and Communication Engineering, Galgotias College of Engineering and Technology, Greater Noida, Uttar Pradesh 201310 India; 3 Department of Electronics and Communication Engineering, Thapar Institute of Engineering and Technology, Patiala, Punjab 147004 India; 4 Manipal Institute of Technology, Manipal Academy of Higher Education, Manipal, India

**Keywords:** Engineering, Mathematics and computing

## Abstract

In controlled dynamic laboratory conditions representative of real-world variability, the gases are usually mixtures rather than individual components. In chemometrics and electronic-nose (E-nose) systems, accurate concentration is a key challenge. Standard algorithms like support vector machine (SVM), k-nearest neighbors (KNN), and shallow multi-layer perceptron (SMLP) have limited feature extraction and poor generalization ability when faced with overlapping responses from sensors, as well as strong cross-gas interactions. While deep learning methods offer enhanced performance, they generally rely on large labeled datasets and cannot inherently maintain robustness in mixed gas scenarios. In order to overcome this limitation, the paper presents a hybrid approach of 3D convolutional neural networks (3DCNNs) with semi-supervised generative adversarial network (SGAN). The 3DCNN component captures spatiotemporal dynamics of sensor array responses, while the SGAN improves generalization under limited labeled data by generating realistic synthetic samples. Experimental results demonstrated that the model obtains a classification accuracy of 99.10%, which is higher than SVM (93.80%), KNN (92.60%), and SMLP (95.30%). These results indicate that the model can be well used for high-precision gas mixture analysis. This work supports SDG 9 (Industry, Innovation and Infrastructure) through advanced AI-based gas sensing, SDG 11 (Sustainable Cities and Communities) by enabling accurate air-quality monitoring, and SDG 3 (Good Health and Well-Being) by improving detection of hazardous gas mixtures that impact public health.

## Introduction

Detecting and identifying gases has a wide range of applications, such as in the chemical industry^1^, food^2^, air^3^, and medical^4^. Electronic nose (E-nose) systems have been developed, inspired by the biological sense of smell, that can be used to monitor atmospheric pollution^5^, assist in medical diagnoses^6^, ensure food quality^7–9^, and enhance public safety^10^. E-nose systems are available commercially. However, they are deficient from a technological maturity and performance point of view when compared with more established sensing paradigms like machine vision and machine hearing^11^. The lack of feasible miniaturization can mostly be attributed to intrinsic limits of sensor materials and manufacturing processes, giving rise to poor selectivity, limited repeatability, and long-term stability^12,13^.

Significant advances were made in the development of high-performance sensors with improved sensitivity and selectivity in controlled laboratory environments, however, cross-sensitivity to chemically similar gases is still a major hurdle, hampering large-scale deployment and commercialization^14^. The performance of sensors is usually characterized in terms of statistical limit of detection, limit of quantification, and sensitivity^15,16^.

Precise monitoring of gas concentration proves especially crucial in sectors such as petroleum, chemical, metallurgical mining, and environmental engineering that experience the risk of severe explosions and toxic gases^17,18^. Real-time gas concentration measuring systems, which have been deployed extensively along with sensor deployment, remain descriptive overall and give no predicted values, only current or historical values. As a result, they provide little help for preventing mishaps. With the use of intelligent prediction methods^19–21^, data-driven digital models can be created based on historical measurements, which can also help in extrapolating trends of gas concentrations and forecasting in a better way^22^. Predictive insights are valuable to anticipate potential risks and accidents and develop strategies to avert them^23^.

Convolutional neural networks (CNNs) are one of the most powerful deep learning architectures from a data representation point of view. CNN could automatically learn and extract important image features by itself without going through feature engineering. Through using local connectivity and weight-sharing, CNNs reduce the unnecessary complexity of the model and mitigate overfitting. So CNNs are effective for the high-dimensionality of sensor data. As deep learning continues to gain acceptance in gas sensing, it was noted that CNN-based models have shown promising performance in identifying mixed gas and estimating concentration^24–28^.

Many of the existing CNN-based methods in gas detection use supervised learning with the sensor array response data used mostly for classification^29,30^. Nevertheless, these conditions are often overlooked in testing, even though they are important for mixing. Furthermore, previous research has been confined to specific cases of binary gas mixtures at a few concentration levels, such as ethylene-carbon monoxide or ethylene-methane mixtures. Even though they show that gas recognition based on CNNs is doable, this does not address the more involved, real-world problem of mixed-gas analysis. Here, the gas type and concentration are highly non-linear and dependent.

In this paper, the joint problem of qualitative and quantitative identification of mixed gases under dynamic conditions is tackled. We put forth a framework based on semi-supervised generative adversarial networks (SGAN) that effectively disentangles gas type concentration interference for better recognition accuracy and generalization performance. The suggested methodology uses semi-supervised learning, which allows the use of both labeled and unlabeled data to provide the classification element with strength as well as generate concentration estimates that are more reliable, especially where few labeled examples are involved. This methodology has specific relevance in practice as gas mixtures like ethylene, carbon monoxide, or methane occur in practice in uneven and constantly varying concentration ratios. The main contributions of this work can be summarized as follows:

- Integration of CNNs with dynamic gas testing techniques, moving beyond static concentration classification.

- Introduction of a SGAN framework that effectively leverages unlabeled data to mitigate label scarcity in gas sensing applications.
- Simultaneous qualitative and quantitative identification of mixed gases, addressing nonlinear interference between gas type and concentration.
- Improved recognition accuracy and generalization performance compared with traditional CNN-based and classical machine learning (ML) approaches.
- Establishment of a scalable and robust framework suitable for real-world gas mixture monitoring under dynamic conditions.

To the best of the author’s knowledge, this work for the first time integrates a 3DCNN with a SGAN for simultaneous qualitative and quantitative analysis of mixed gases, enabling robust classification and concentration estimation even under limited labeled data conditions.

## Literature review

Chemi-resistive gas sensors have gained a lot of attention for their simple structure, high sensitivity, and fast response time. These attributes make them suitable for various applications. Among them, the detection of volatile organic compounds (VOCs) like ethyl alcohol is a very useful application^31^. Similarly, another useful application is their use in breath analysis and point-of-care diagnosis. Most of these sensors are metal oxide-based devices that are compact in size and are cheap and easy to use. Moreover, their sensing performance is also quite good. Having this advantage, however, their real-world application becomes difficult due to overlapping and masking effects of interfering species in multi-component gases. Difficult electrical responses are generated by these cross-sensitivities that are hard to separate due to the presence of multiple gases. Therefore, single-sensor solutions cannot be trusted. Correspondingly, real-world tests across large scales cannot be carried out.

The limited scope of chemical detection by analytical sensor systems can be augmented by utilizing more sensor systems to respond to pattern data from gas mixtures. The E-nose system utilizes an array of two or more sensors rather than the specific two sensors. E-nose systems that use ML algorithms in combination with these responses help in improved discrimination and quantification of gas mixtures^32^. Most of the earlier E-nose studies utilized classical ML techniques. These include support vector machines (SVMs)^33^, k-nearest neighbors (KNN)^34^, and stepwise multilayer perceptrons (SMLP)^18^. Further, these techniques are useful in gas identification and concentration estimation. To enhance the selectivity and sensitivity of gas sensor arrays, authors in^35^ employed feed-forward neural networks (FFNNs) trained by Levenberg-Marquardt (LM) and Bayesian regularization (BR) algorithms. By using a dataset that requires no prior permission and consists of 16 sensors exposed to C_2_H_4_–CO and CH_4_–C_2_H_4_ mixtures, they can successfully discriminate and estimate concentrations. Although both training strategies have achieved good results, the BR-based one has higher accuracy but is more complex. The authors in^35^ made additional contributions by introducing a tree-based ML framework that will result in faster and more robust mixed-gas identification.

Regular pattern recognition techniques calculate the features manually. This process takes up a lot of time. Also, it highly depends on the application. Deep learning methods need not rely on heuristics or handcrafted features. Rather, they learn the mapping from raw sensor input to high-level representation through end-to-end training. Recurrent neural networks (RNNs), including long short-term memory (LSTM) networks and gated recurrent units (GRUs), have been used to model temporal dependencies and causal relationships in sequential data by maintaining and updating hidden states over time. In E-nose applications, models based on LSTM have proved to be effective in fields such as beef quality monitoring^36^, sensor drift compensation, and gas concentration prediction^37^. As gas mixtures are complex, CNNs that prove to be powerful nonlinear models with good feature extraction are being investigated.

The average recognition accuracy of deep CNN (DCNN) architectures for gas mixtures, which contain C_2_H_4_, CO, and CH_4_, is obtained as 96.15%^38^. Hybrid models based on deep learning, using CNNs and extreme learning machines, are capable of detecting anomalous odors in real time, with an accuracy of more than 92%^39^. CNN classifiers can achieve mixed-gas recognition rates of about 81.7%^40^ due to the image-based portrayals of sensor responses. More sophisticated architectures such as SimResNet and deeper CNNs with a parameter specification of 38 layers have 95% and 95.2% classification accuracy, respectively^41,42^.

The conventional CNN-based models, however, are not necessarily effective at dealing with the sequential dynamic nature of gas sensor responses, which are time-evolving signals that will undergo continuous changes as time passes and are also affected by the sensor cross-sensitivity. The long-term temporal dependency is limited in CNNs, while they are good at spatial feature extraction. RNN-based models are able to partly deal with this issue by performing a recursive processing on the time-dependent signals in a manner similar to biological neural systems^43,44^. Tasks like speech recognition and emotion analysis have been successfully handled by such models^42^. However, gas sensor data presents distinct challenges. Individual sensors exhibit heterogeneous temporal response characteristics, while cross-sensitivity effects introduce strong interdependencies among sensor channels. These factors complicate both the qualitative interpretation and quantitative analysis of the sensor signals. But gas sensor data is different. This is where each sensor exhibits different temporal response characteristics, and cross-sensitivity causes strong interdependencies among sensors, which upsets their qualitative and quantitative analysis.

These limitations required architectures capable of modeling the spatial correlations across the sensor arrays and the temporal dynamics of the gas interactions together, especially when labeled data is scarce. This observation inspires the combination of spatiotemporal deep learning models with semi-supervised learning techniques for effective identification and concentration estimation of mixed gases.

## Materials and methods

Integrating a 3D CNN with a SGAN enables robust spatiotemporal feature learning for gas concentration estimation. Here, the raw time-series responses of the sensor array are used for training and evaluation. This allows the model to capture spatial correlations between sensors and temporal dynamics of gas interactions. To evaluate the approach, we performed extensive experiments on the publicly available Gas Sensor Array under Dynamic Gas Mixtures dataset^45^.

### Dataset

**Fig. 1:**
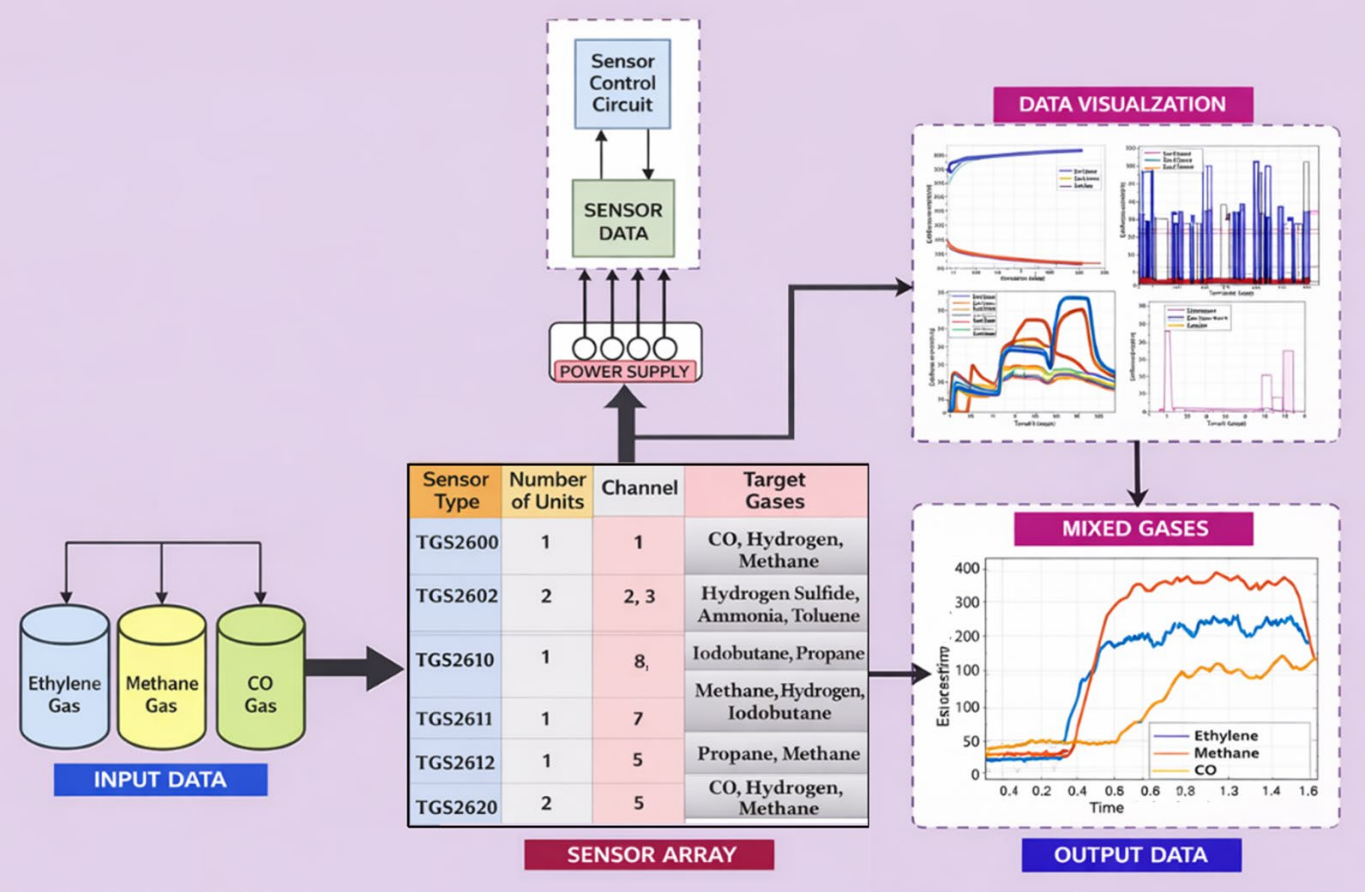
Data acquisition and visualization pipeline of the open-source MOX gas sensor dataset^45^.

The dataset includes continuous measurements of two binary gas mixtures, which are C_2_H_4_–CH_4_ and C_2_H_4_–CO, made in ambient air. Each experiment used a total of 16 sensors, which operated continuously for 12 h. The gas concentration levels for both mixtures were regulated using a single protocol. A computer controlled the batch mixing to ensure that the concentration profiles were generated accurately and reproducibly. As shown in Fig. 1, the setup of the experiment consists of a data acquisition system, a power control unit, and a chemical delivery system.

The sensor array incorporates 16 commercial metal-oxide sensors (TGS-2600, TGS-2602, TGS-2610, and TGS-2620), which are connected to custom signal-conditioning circuitry. All sensors were turned on at 5 V, and the measurement chamber was maintained at 60 mL with a flow rate of 300 mL/min using a mixture of gases. Each gas was selected at a concentration that produced comparable sensor response magnitudes. It was done with low concentration to avoid the simple pattern recognition.

Laboratory synthesis of gas mixtures was done in a strictly controlled laboratory environment, with a computer-controlled batch mixing protocol that ensures accurate and repeatable concentration profiles. The constant volume of the experimental chamber was 60 ml, and the total gas flow rate was kept at 300 ml/min. All sensors were maintained at a constant heater voltage of 5 V to send uniform thermal conditions throughout the array. The sensor array was a commercial array of sixteen metal-oxide sensors (TGS 2600, TGS 2602, TGS 2610, and TGS 2620) interconnected to custom signal-conditioning circuitry. The duration of each mixture experiment was twelve hours, and the concentration levels were varied among eight equal time intervals with randomized profiles of ppm. C_2_H_4_, CO, and CH_4_ were set at a range of 0–20 ppm, 0-600 ppm, and 0-300 ppm, respectively.

The raw sensor signals were recorded in the continuous mode and then down sampled to 10 Hz, a technique that is used to reduce the redundancy without affecting the important dynamic behavior of the system. The concentration values provided in the dataset were annotated to each of the data samples. The entire data set was further divided into time segments of fixed length and pretrained before normalization. The general data acquisition and visualization pipeline is shown in Fig. 1.

### Proposed hybrid 3DCNN-SGAN network

CNNs have demonstrated exceptional performance in computer vision as well as a number of other fields. 3D-CNN-based architectures have shown significant performance in spatiotemporal feature extraction and complex recognition^46^. Applying a 3D-CNN to extract temporal, frequency, and phase information multi-domain features is a state-of-the-art approach that captures the complex dynamics. This unified extraction strategy simplifies the pipeline and circumvents the loss of accuracy that frequently occurs with individual feature extraction strategies.

In presenting gas-sensor array data, the common methodology usually presents the naturally three-dimensional data, sensor index, time, and response magnitude, as two-dimensional time-sensor plots. Nevertheless, the dimensionality reduction in such a way masks the fact that the measurements are more spatiotemporal in nature. In mixed-gas conditions, the cross-sensitivities cause coupled spatial and temporal dependencies, such that inter-sensor correlations and temporal dynamics co-evolve in a joint way which cannot be sufficiently modeled by a simple two-dimensional representation.

In order to overcome this weakness, a 3DCNN is proposed. This architecture simultaneously captures: (i) the spatial covariance across the sensor array, (ii) the time dynamic of gas sensor interactions, and (iii) the local spatiotemporal motifs of moving concentration patterns. The convolution kernels of a 3DCNN move through sensor, time, and feature space simultaneously, unlike one-dimensional CNNs, which are limited to the time axis, or two-dimensional CNNs, which are essentially limited to the sensor or time axis. This coherent spatiotemporal filtering enables the network to recognize response dynamics associated with concentration and complex sensor interaction patterns that cannot be identified with separable 1D or 2D kernels.

In turn, the implementation of a 3DCNN provides an architectural benefit of mixed-gas analysis. Here, not only the temporal history of exposure but also the co-occurrence and relative concentration of several gases determine the response of each sensor, a dependence that is also encoded inherently in the volumetric convolutional model.

The hybrid 3DCNN-SGAN network presented for mixed-gas sensor detection combines the power of a 3D CNN and a supervised generative adversarial network to improve accuracy, robustness, and generalization performance in gas classification and concentration estimation.

The 3DCNN is a powerful ML feature extractor that takes in multichannel sensor time-series data to represent gas concentration changes. The gas sensor can learn both spatial and temporal dependencies with the sensor signals, capturing the mixed gas relationships that cannot be modelled by using traditional methods. The representations that have been extracted are fed to the SGAN, where the generator creates synthetic sensor patterns, similar to real measurements, which augment the training data. The discriminator is a supervised model that conducts real-fake differentiation and disparity classification of gas type concentration.

Through adversarial training, the generator continually produces more realistic samples, which in turn progressively improves the detection and classification performance of the discriminator. This collaboration makes features stronger. Also, it makes the model more sensitive, selective, and adaptable to changing gas environments.

**Fig. 2:**
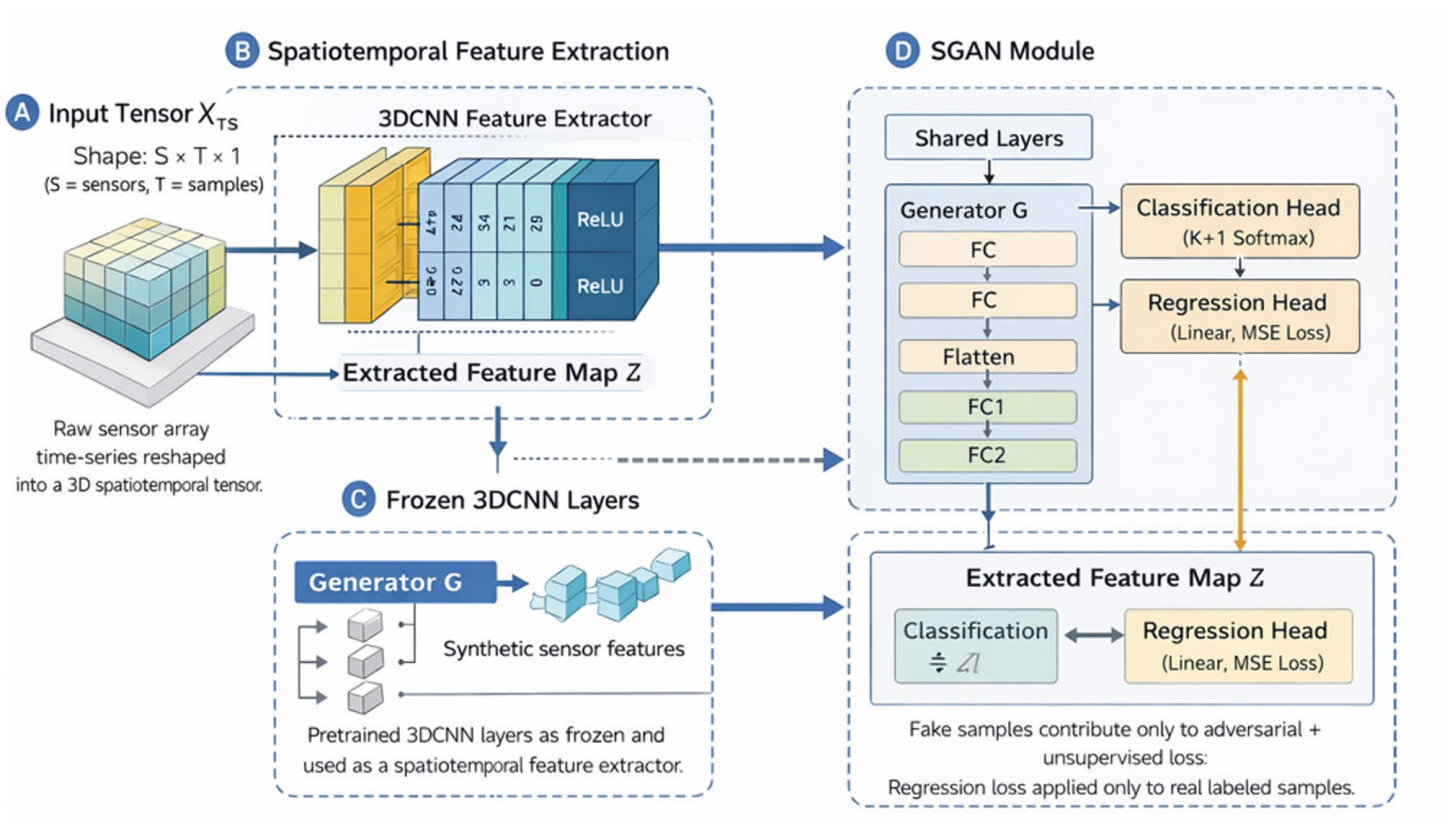
Architecture of the proposed hybrid 3DCNN-SGAN framework.

The complete architecture, as illustrated in Fig. 2, comprises 3D convolution, pooling, fully connected, and regression layers. The two main components of training focus on spatiotemporal feature extraction and 3D-CNN optimization. For this purpose, stacked SGAN modules are used to refine the concentration prediction via semi-supervised learning. This generates realistic sensor response and robustness across a wide range of datasets.

**Spatiotemporal Feature Extraction**: Let $X\in {\mathbb{R}}^{S\times T}$ denote the raw time-series sensor array response, where $S$ is the number of sensors and $T$ is the number of temporal samples per instance. Each column of $X$ corresponds to a sensor response vector at a given time step. To enable spatiotemporal convolution, the 2D input matrix $X$ is reshaped into a 3D tensor ${X}_{TS}\in{\mathbb{R}}^{S\times T\times 1}$, where the third dimension represents a single input channel.

The symbol ${X}_{c}$ denotes the output feature maps obtained after applying a 3D convolution operation to ${X}_{TS}$. These feature maps represent local spatiotemporal patterns across sensors and time. The final extracted representation is then flattened and passed to the fully connected layers for regression and classification. The pattern recognition problem involves predicting target gas concentrations $\left(y\in R\right)$ from input time series ${X}_{T}^{S}$, where *S* ($=1,2,\dots.N$) is the number of sensors in the array and *T* denotes the length of the time series (*t*). Given that the sampling frequency is ${f}_{s}$ and the concentration transition time is ${t}_{c}$, the concentration level can be calculated accordingly. Each concentration level corresponds to ${T}_{c}={t}_{c}\times {f}_{s}$ time instances. It is called an instance when ${X}_{c}$ is ${T}_{c}\times S$ and $({X}_{c};y)$ is a tuple. While modeling gas concentrations, *X* represents time series of sensor array responses, and *Y* represents gas concentrations. There will be one input channel and a 3D tuple input to the network. Due to the spatial and temporal correlation between each sensor in the array, the sensor array response is reshaped from 2D to 3D data ${X}_{Tc}^{S}$, with size $X[m,n,t]$. A function map and kernel have depth parameters, so the convolution also has to move in time. As part of CNN reconstruction, 3D convolutions are carried out to evaluate attributes in both spatial and temporal dimensions (*m* × *n*). A convolution layer encapsulates temporal information by binding its function maps to contiguous frames of the preceding layer.

First, the 3DCNN network is trained as a regression network in order to save the model parameters for later use as a feature extractor. As shown in Fig. 2, the 3DCNN network is trained using the initial batch of data, and then the 3D convolution layers and pooling layers are frozen to obtain spatiotemporal features from subsequent batches. A convolution stage combines convolution and activation operations. There are eight layers in the regression network, starting with the input layer and ending with the output layer. Additionally, there are two convolution layers, two pools, and two fully connected (FC) layers. It alternately added convolutional layers and pooling layers. A layer of FC is placed behind a layer of pooling. An orthographic projection is done over a 1D field vector to create the pooling layer’s 3D field map. The following formula can be used to calculate the output of a convolutional 3D layer:

1 $${C}_{i,j}^{m,n,t}=tanh({b}_{ij}+\sum_{k}\sum_{x=0}^{{X}_{i}-1}\sum_{y=0}^{{y}_{i}-1}\sum_{z=0}^{{Z}_{i}-1}\times{w}_{ijk}^{xyz}{C}_{\left(i-1\right)k}^{\left(m+x\right)\left(n+y\right)\left(t+z\right)},$$

where $m,n,\,\mathrm{and}\,t$ denote the spatial (sensor), spatial (feature), and temporal indices of the output feature map, respectively. The term $K$ represents the size of the 3D convolution kernel along the temporal dimension. The weights ${w}_{i,j,k}$ correspond to the kernel parameters connecting the $l$^*th*^ feature map in the previous layer to the current layer, and $b$ is the bias term. The summation is performed over all kernel positions and input channels.

Using Eq. (1), the value at the location (*m*, *n*, *t*) on the ${i}^{th}$ layer of the ${j}^{th}$ function map is calculated. In this case, ${Z}_{i}$ is the size of the 3D kernel in time direction. A kernel connected to the ${k}^{th}$ feature map in the prior layer ${w}_{ijk}^{xyz}$ has the coordinates ${(x,\,y,z)}^{th}$ and $b$is the bias.

Using the pooling layer, the input characteristic map is sampled using the input data’s spatial resolution, which is further decreased by the pooling layer. As part of down sampling, features can be removed from the function map in order to maintain robustness and ensure that the displacement and scaling are not degraded. Since sensor responses are continuous and correlated over time, the pooling layer processes values from several contiguous locations. As a result, the down sampled features will be consistent. A dropout process is applied before the $FC1$ layer to improve numerical performance and prevent overfitting during the final FC layers to quantify the gas mixture. Using Eq. (2), the loss function is MSE.

2 $$MSE=\sum_{i=1}^{n}\frac{{\left({y}_{i}-{y}_{i}^{p}\right)}^{2}}{n},$$

where$y$ is the actual concentration set and ${y}^{p}$ is the predicted concentration set. Neurons are used as a regressor to represent the number of gases in the mixture, and linear activation functions are used to represent the amount of the mixture occupied by gases. Hence, the spatiotemporal features are extracted by using the parameters of two sets of 3D convolutions. Sensor data consists of sensor resistance values that vary over time. As a result of the model, the raw data has been reshaped and normalized in order to feed the network in $X[m,\,n,\,t]$ form over time. The method for displaying the visual information of preprocessed data is to convert the data shape to $\left(\left(m\times \sqrt{t}\right),\,n\times \left(\sqrt{\left(t\right)}\right)\right)$ and then concatenate the data in two directions. The output for three filters in the first convolution layer of the network with 15 filters, with a kernel size of 3 × 3. There are 10 filters with the same kernel size in the second convolution layer.

**Supervised Generative Adversarial Network (SGAN)**: If there are only a few labelled data points but a lot of unlabelled data points, this approach is especially useful. There are two types of GANs: Generators (*G*) and processors (*P*). It starts with random noise and transforms it into data that could resemble the real dataset. An identifier for discriminating between real data (from the dataset) and synthetic data (from the generator) is the discriminator (*D*).

In the proposed architecture, the discriminator is extended to support both classification and regression tasks. Specifically, it consists of two output heads: (i) a $\left(K+1\right)$-class softmax classification head for gas-type discrimination (including the synthetic “fake” class) and (ii) a separate regression head for continuous gas concentration estimation.

The regression head is trained using a mean-squared error (MSE) loss applied only to real labeled samples. Synthetic samples generated by the generator contribute exclusively to the adversarial and unsupervised classification loss and are not assigned concentration labels.

The total training objective is defined as:

3 $${\mathcal{L}}_{total}={\mathcal{L}}_{sup}^{cls}+{\lambda}_{u}{\mathcal{L}}_{unsup}+{\lambda}_{r}{\mathcal{L}}_{reg},$$

where ${\mathcal{L}}_{sup}^{cls}$ is the supervised classification loss, ${\mathcal{L}}_{unsup}$ is the unsupervised adversarial loss, ${\mathcal{L}}_{reg}$ is the regression loss for concentration estimation, and ${\lambda}_{u}$, ${\lambda}_{r}$ are weighting coefficients.

This formulation resolves the apparent contradiction between discrete SGAN classification and continuous concentration prediction and enables simultaneous qualitative and quantitative analysis within a unified semi-supervised framework.

SGANs generate synthetic sensor readings that mimic the real ones. In the proposed model, various gas mixtures at different concentrations are simulated using random noise. A standard classifier is used to classify a data point $x$ into one of $K$ possible classes. This model takes in a *K*-dimensional vector of logits $\{{l}_{1},\ldots,{l}_{K}\}$, as input and computes the class probability distribution using the SoftMax function: ${P}_{model}\left(y=j|x\right)=\frac{exp\left({l}_{j}\right)}{\sum_{K=1}^{K}exp\left({l}_{k}\right)}$. An observed label is then combined with the predicted distribution ${P}_{model}\left(y\right|x)$ in supervised learning by minimizing cross-entropy. Using any standard classifier, the semi-supervised learning is applied with a GAN generator $G$, labeling with the newgenerated class $Y=K+1$, and correspondingly increasing the dimension of the classifier output. Using ${P}_{model}\left(y=K+1|x\right)$, the probability of the original GAN being fake, which corresponds to $1-D\left(x\right)$. When half of the data set is generated and half is real, the loss function for training a classifier is given as,

4 $$L=-{E}_{x,y\sim{P}_{data}(x,y)}\left[{log}\,{p}_{model}(y|x)\right]-{E}_{x\sim G}\left[{log}\,{P}_{model}(y=K+1|x)\right]$$

5 $$L={L}_{supervised}+{L}_{unsupervised}$$

where

6 $$\begin{aligned}{L}_{supervised}&=-{E}_{x,y\sim{p}_{data}\left(x,y\right)}{log}\,{p}_{model}\left(y|x,y<K+1\right),\\ {L}_{unsupervised}&=-\left\{{E}_{x\sim{p}_{data}(x)}{log}\left[1-{p}_{model}(y=K+1|x)\right]\right.\\ &\quad\left.+{E}_{x\sim G}Log\left[{P}_{model}\left(y=K+1|x\right)\right]\right\}\end{aligned}$$

The total cross-entropy loss was decomposed into the standard supervised loss function ${L}_{supervised}$ (the negative log probability of the label given the real data) and the unsupervised loss ${L}_{unsupervised}$, which is essentially the standard GAN game value when $D\left(x\right)=1-{p}_{model}(y=K+1|x)$ is substituted into the expression.

7 $${L}_{unsupervised}=-\left\{{E}_{x\sim{p}_{data}(x)}log\,D(x)+{E}_{z\sim noise}\,\mathrm{log}\,\left(1-D\left(G\left(z\right)\right)\right)\right\}$$

If $c\left(x\right)$ is an undetermined scaling function, then $\mathrm{exp}\left[{l}_{j}\left(x\right)\right]=c\left(x\right)p\left(y=j,x\right)\forall\, j<K+1$ and $\mathrm{exp}\left[{l}_{K+1}\left(x\right)\right]=c\left(x\right)pG\left(x\right)$ are the optimal solutions for minimizing both ${L}_{supervised}$ and ${L}_{unsupervised}$. Unsupervised loss is therefore consistent with supervised loss, and its optimal solution is estimated by minimizing both loss functions simultaneously. In practice, ${L}_{unsupervised}$ will only be helpful if the proposed classifier requires training *G* to approximate the data distribution, which isn’t trivial to minimize. Using the classifier’s discriminator *D*, *G* can be trained to minimize the GAN game-value. It is found that optimizing *G* with feature matching GAN is very effective for semi-supervised learning, whereas training *G* with mini-batch discrimination does not work at all.

Also, note that the $K+1$ output classifier is over-parameterized: setting ${l}_{j}\left(x\right)\leftarrow {l}_{j}\left(x\right)-f\left(x\right){\forall}_{j}$ does not change the output of the SoftMax when $f\left(x\right)$ is subtracted from each output logit. If ${l}_{K+1}\left(x\right)=0\forall\,x$ is equivalent to ${L}_{supervised}$, then the original classifier with $K$ classes’ supervised loss function is ${L}_{supervised}$, while the discriminator $D$ is $D\left(x\right)=\frac{Z\left(x\right)}{Z\left(x\right)+1}$, where $Z(x)=\sum_{k=1}^{K}\mathrm{exp}\left[{l}_{k}(x)\right]$ is the original classifier’s supervised loss function. Compared with traditional preprocessing approaches such as PCA or ICA followed by a supervised classifier, the proposed SGAN framework offers clear analytical advantages for mixed-gas separation. First, the unsupervised loss component explicitly drives the decision boundary into low-density regions of the data distribution, thereby enlarging the geometric margin between gas classes and reducing classification error. Second, because the generator synthesizes sensor responses across varying concentrations, the discriminator learns features that retain gas-type information while discarding concentration as a nuisance variable, leading to concentration-invariant representations.

**Fig. 3:**
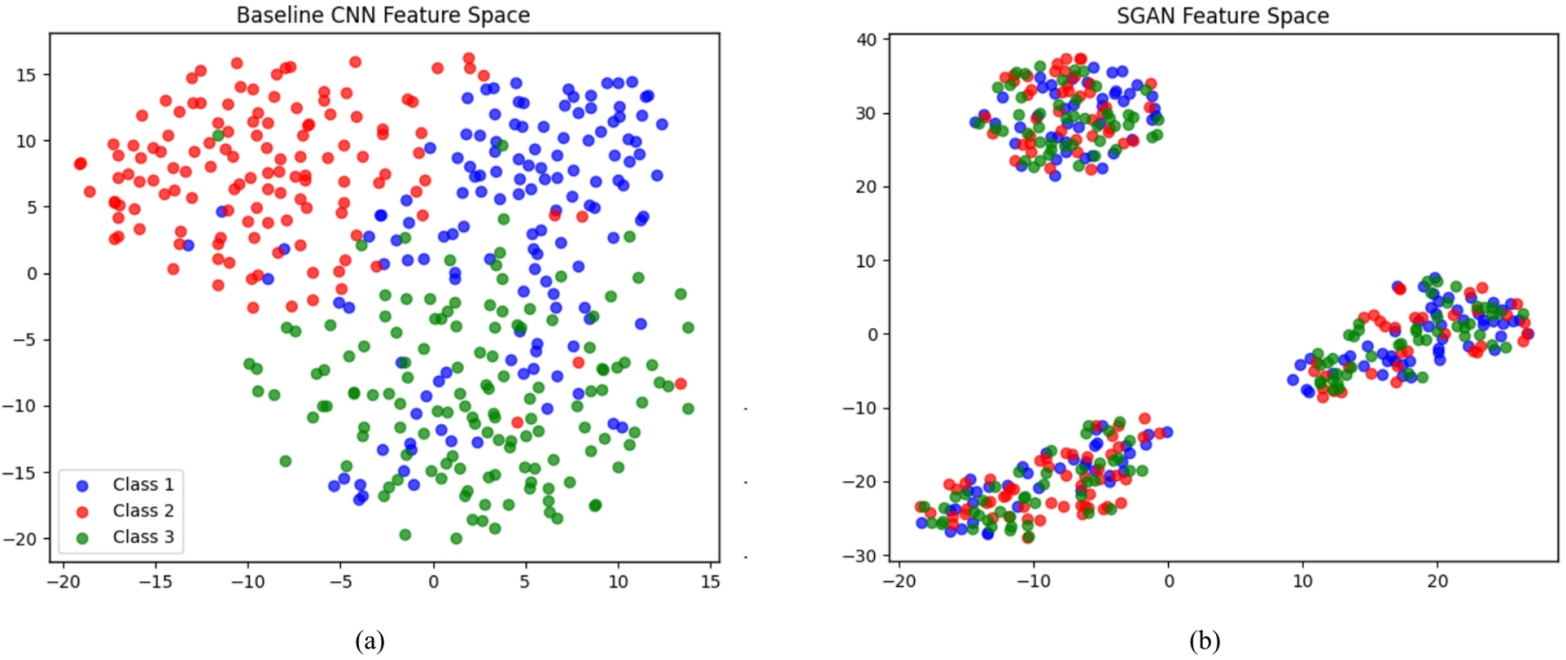
Feature space visualization using t-SNE: (**a**) Baseline CNN produces overlapping clusters for gas mixtures, showing limited separability, (**b**) The proposed SGAN yields tighter and more distinct clusters, demonstrating improved discrimination between gas classes. Class 1 = Ethylene-CO, Class 2 = Ethylene-CH_4_, Class 3 = Other mixtures.

Third, this mechanism reduces the class conditional overlap of mixtures, e.g., ethylene-CO and ethylene-CH_4_, thus directly maximizing separability and reducing the Bayes error bound. Through the addition of the training space with realistic samples, which are labeled with consistent labels, SGAN essentially expands the sample population and reduces the variance of parameter estimation, which traditional preprocessing pipelines cannot achieve. All of these characteristics show that SGAN has a mathematical and practical advantage over traditional preprocessing methods of qualitative and quantitative gas mixture recognition.

In order to further demonstrate the merits of the proposed SGAN framework, we visualize the acquired feature representations by using t-SNE (Fig. 3). The clusters of different gas mixtures in the baseline CNN (Fig. 3a) are strongly overlapping, particularly between the ethylene-CO and ethylene-CH_4_, which indicates that they cannot be separated well in the latent space. By contrast, the SGAN feature space (Fig. 3b) shows tighter, more compact clusters with clearer boundaries between classes, demonstrating that the generative-discriminative training strategy enhances class separability and improves discrimination performance.

## Results and discussion

**Fig. 4:**
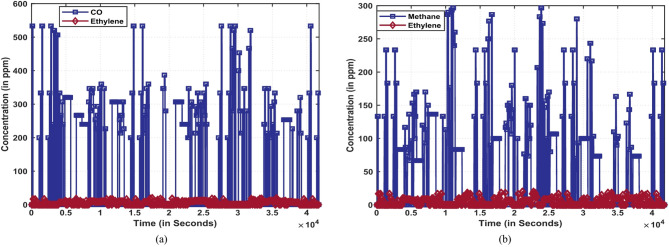
Concentration of mixed gas sensor data: (**a**) Binary gas mixtures of ethylene-CO, (**b**) Binary gas mixtures of ethylene-methane.

In this section, the proposed hybrid 3DCNN-SGAN network is elaborated. In order to verify the effectiveness of the proposed model for mixture gas classification, extensive experiments were conducted with the publicly available dataset. Figure 4 illustrates the concentration levels of mixed gas sensor data for two distinct binary gas mixtures: Ethylene-CO (in Fig. 4a) and ethylene-CH_4_ (in Fig. 4b). The dataset is labeled with concentration categories, allowing for precise analysis and classification.

As the raw dataset is labelled in its concentration types with 4,178,504 + 4,208,261 = 8,386,765 instances, where 4,178,504 represents the instances between C_2_H_4_ and CH_4_ and 4,208,261 represents the instances between C_2_H_4_ and CO, the category was manually labelled in the manner of multi-label and split the raw dataset into 593 samples that represent the same class, respectively. Furthermore, the dataset was sampled down to 10 Hz, and the whole sample was randomly split into 80% training and 20% testing sets.

**Fig. 5:**
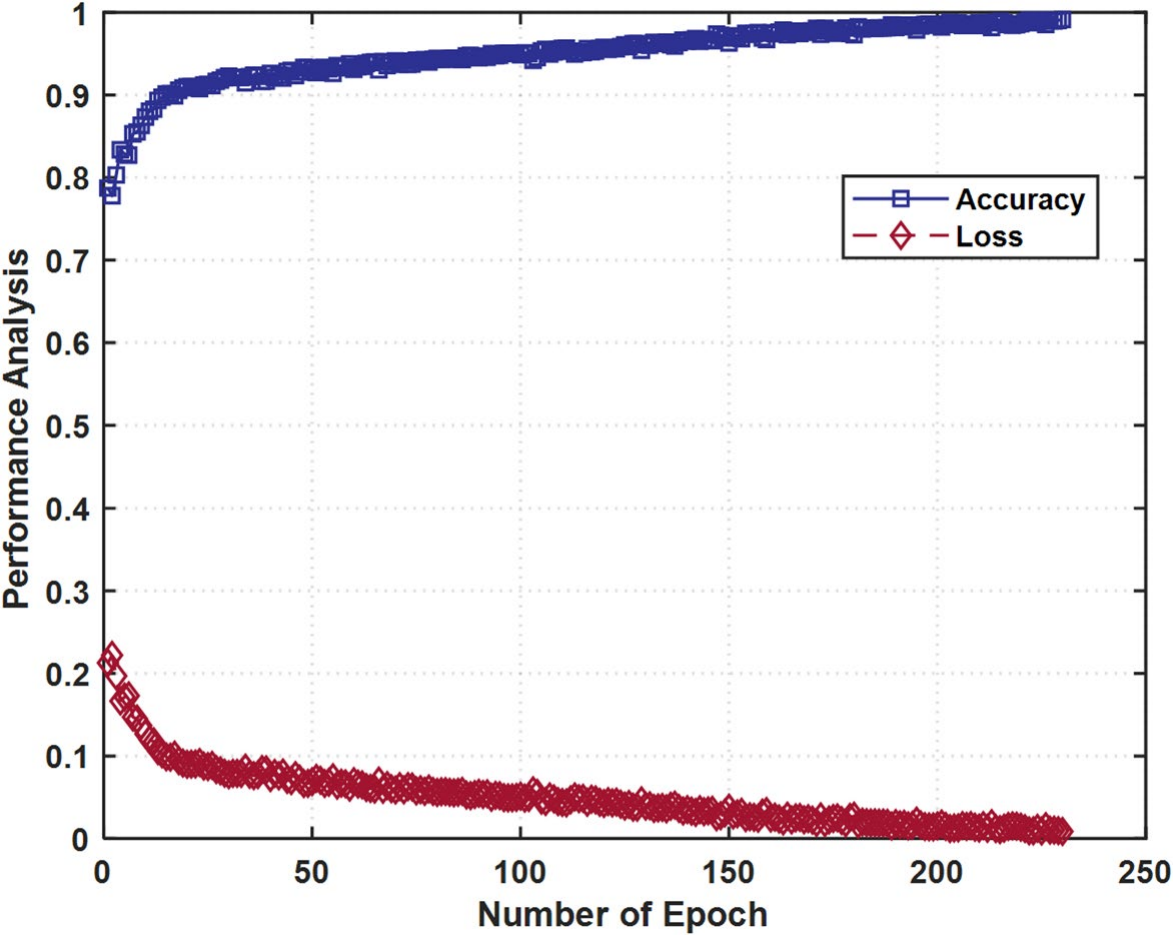
Performance analysis of training accuracy and loss versus number of epoch.

The performance of the 3DCNN-SGAN model is assessed using accuracy and loss metrics on the training dataset. A high accuracy corresponds to a low loss, indicating better model performance. The blue curve represents accuracy, which rises rapidly in the initial epochs as the model learns fundamental patterns from the data. After approximately 100 to 150 epochs, the accuracy is 99.10%, indicating that the model has achieved high performance and stabilized. The red curve depicts the loss function, which starts at a significantly high value and shows a significant decrease throughout the initial stages of the training, as the model becomes more and more accurate in making its predictions. The highest level of training accuracy obtained is 99.10%, which is supported by the information in Fig. 5. All these results testify to the effectiveness of the suggested model to reduce both the problem of underfitting and that of overfitting in parallel.

The D1 and D2 datasets are based on the same UCI dynamic gas mixture dataset but have a different train-test split. D1 is a 75: 25 split, and D2 is an 80:20 split; in both cases a five-fold cross-validation strategy will be used to ensure statistically strong evaluation and the reduction of bias in the evaluation of performance.

**Fig. 6:**
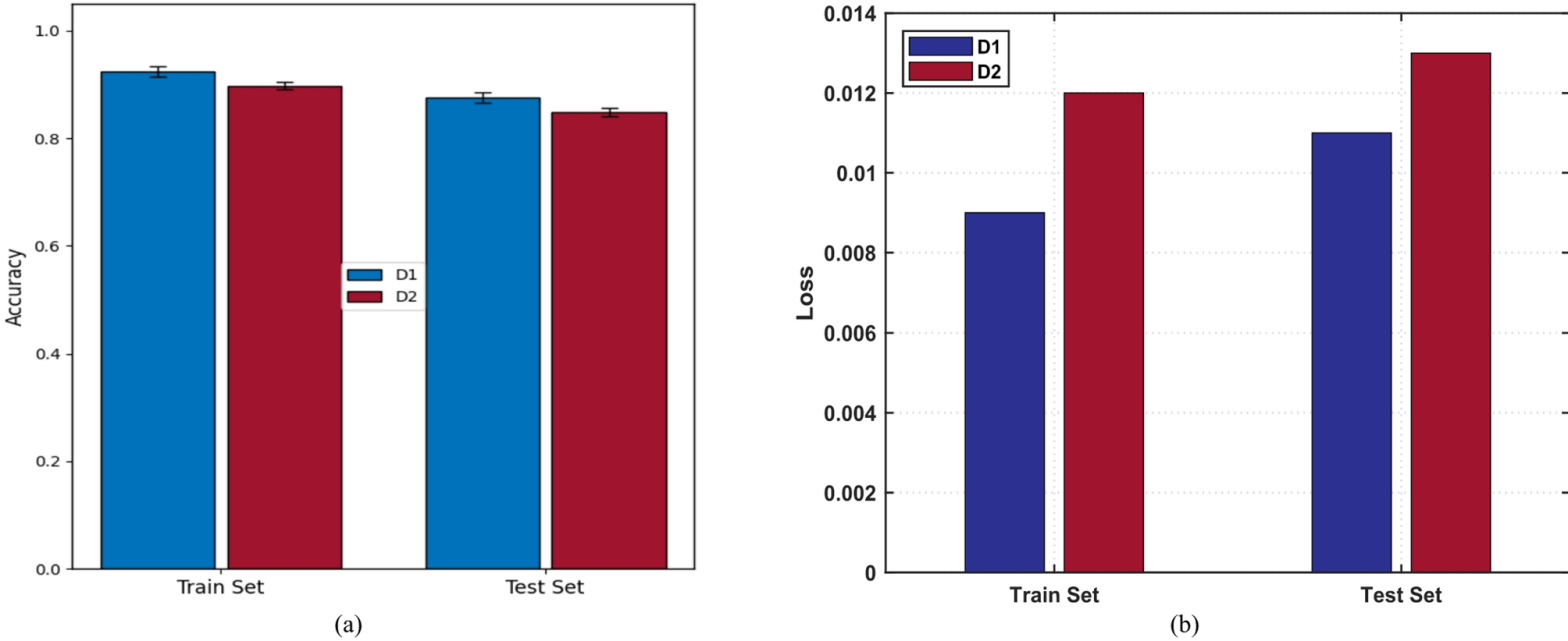
Performance analysis of training accuracy for train and test set: (**a**) Accuracy curves for the train and test sets of D1 and D2, (**b**) Loss curves for the train and test sets of D1 and D2.

The gas detection performance was evaluated using two distinct datasets, D1 and D2, under 5-fold cross-validation to ensure robust and unbiased assessment. The averaged results across folds (Fig. 5) show that both training and testing accuracies stabilize after approximately 150 epochs, with convergence observed consistently across folds. The D1 dataset achieved a mean training accuracy of 0.92 ± 0.01 and a mean test accuracy of 0.88 ± 0.01, while the D2 dataset reached 0.90 ± 0.01 and 0.85 ± 0.01 for training and testing, respectively. These outcomes show that the model is generalizable and is not overfitted to any significant degree, as seen by the fact that training and test accuracy are close to each other. Moreover, the fact that the difference in values was less significant with D1 would suggest that the train-test divide at 75:25 gave rise to a more unbiased data distribution compared to the 80:20 train-test divide used with D2. Figure 6a shows the mean accuracy curves of cross-validation folds in both datasets, and Fig. 6b shows the corresponding loss curves. The fact that both accuracy and loss measures converged to roughly epoch 150 is evidence that the proposed architecture is repeatable and reliable in stabilizing in a high-performance regime on repeated trials.

**Fig. 7:**
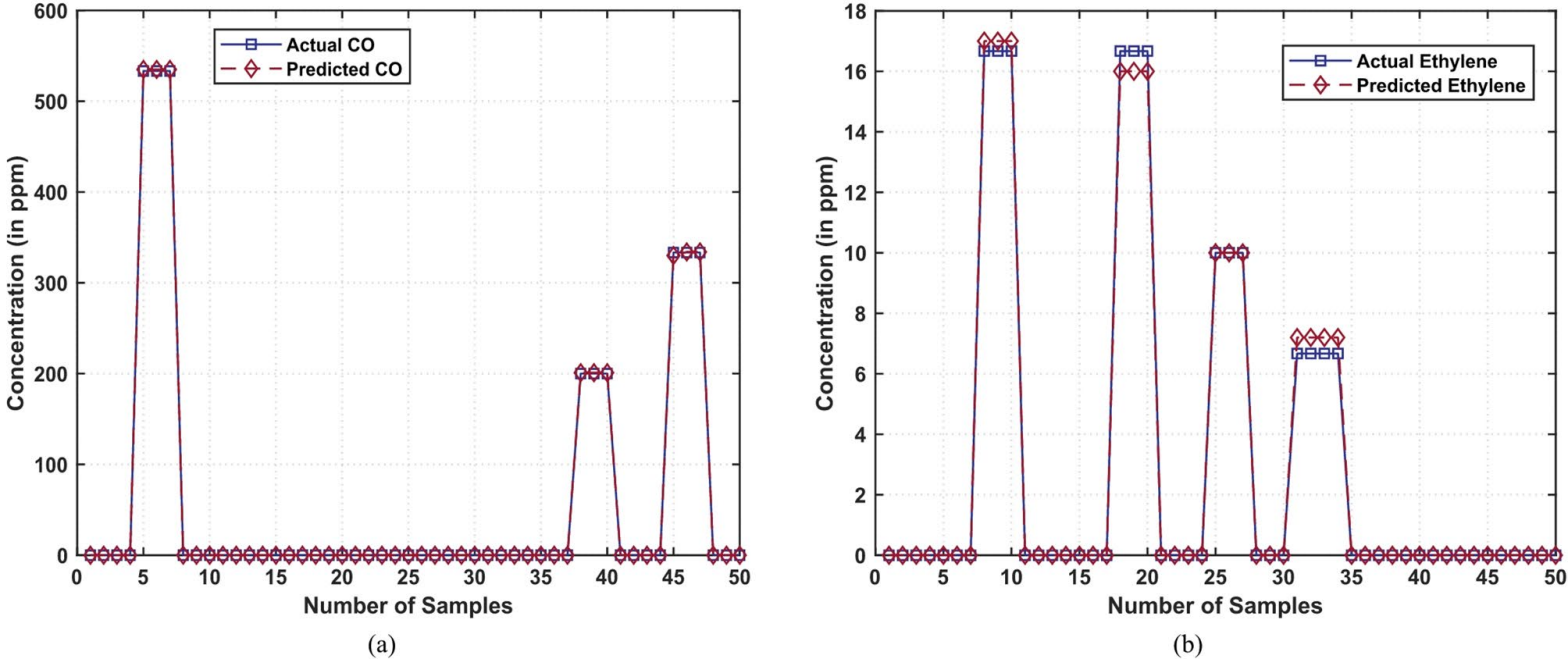
Actual and prediction of CO and C_2_H_4_ concentration (in ppm) in the C_2_H_4_–CO gas mixture dataset: (**a**) Actual and predicted concentrations of CO, (**b**) Actual and predicted concentrations of ethylene.

**Fig. 8:**
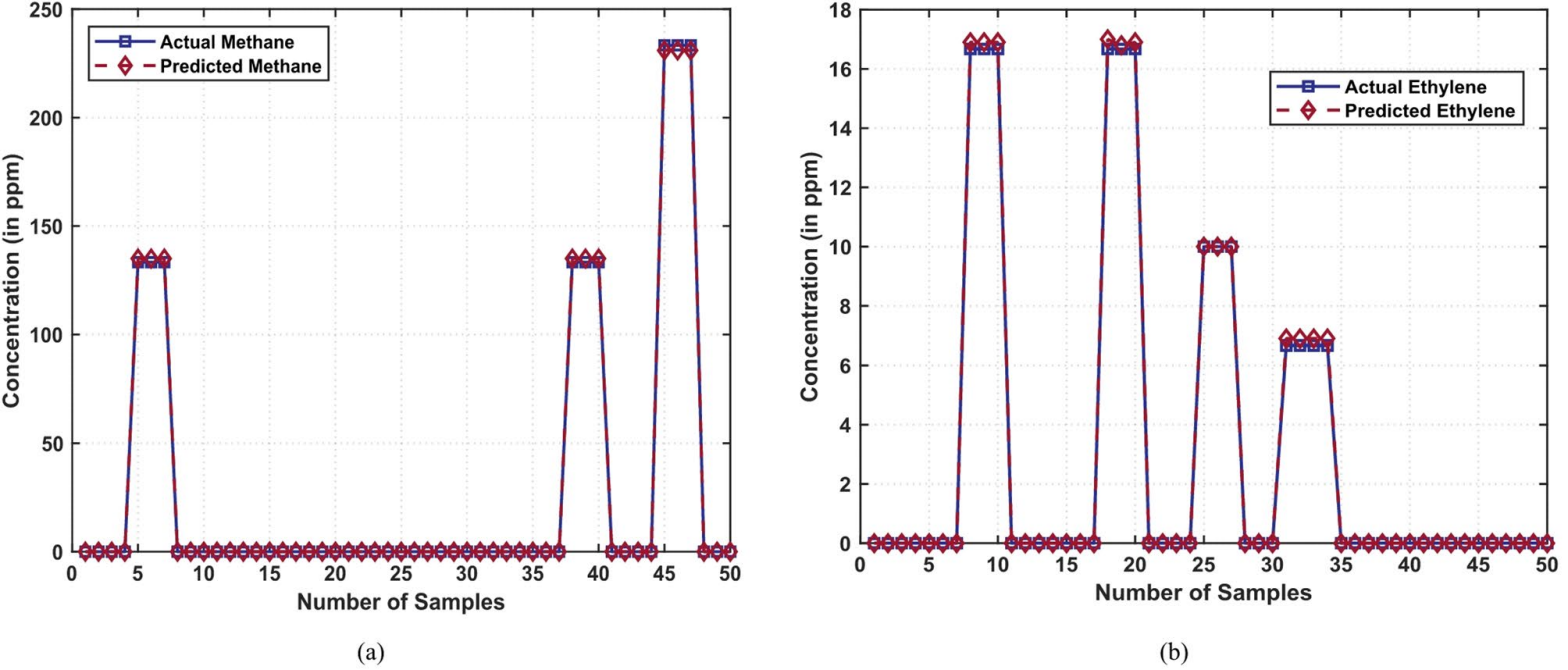
Actual and prediction of CH_4_ and C_2_H_4_ concentration (in ppm) in the C_2_H_4_–CH_4_ gas mixture dataset: (**a**) Actual and predicted concentrations of methane, (**b**) Actual and predicted concentrations of ethylene.

The model has been trained and tested using two different gas mixtures with the predicted concentrations being plotted in Figs. 7 and 8, respectively. Figure 7 shows the real and modelled CO concentration (see Fig. 7a) and ethylene concentration (see Fig. 7b) in an ethylene-CO mixture, measured at 50 random test cases. The actual values are drawn using blue color line, but the predictions are drawn using red color. Similarly, the true and estimated concentrations of ethylene at the same mixture are plotted using a blue and red line respectively. The high similarity between the two curves testifies the high level of precision of the model used to predict CO concentrations.

Figure 8a represents the experimental and the model-based concentrations of methane in a mixed ethylene-methane reservoir. The blue curve denotes the empirical values, and the red curve is the predictions of the model. The fact that the traces intersect the horizontal line of these traces almost perfectly with fifty randomly selected test specimens is a testament to the high degree of accuracy and strength the model has in predicting the concentration of methane even under the conditions of fluctuating concentration ranges. In Fig. 8b, the empirical and predicted ethylene concentrations in the ethylene-methane mixture were presented. Once again, the blue line represents the real measurements, whereas the red line represents the predicted measurements. The near-ideal overlap of the traces is a testament to the high generalizability (and accuracy) of the model in the determination of ethylene concentrations, regardless of the complexity or stochastics of the test samples themselves.

To determine the impact of the semi-supervised component, we introduced a purely supervised 3DCNN baseline, which resembles the structure of the one proposed, except that it lacks the SGAN module. It has been empirically demonstrated that the supervised 3DCNN made less classification accuracy and higher regression errors than the 3DCNN-SGAN setting, which also supports the hypothesis that generative augmentation, together with semi-supervised learning, provides significant gains in terms of generalization and robustness in data settings with sparsely labeled samples.

**Fig. 9:**
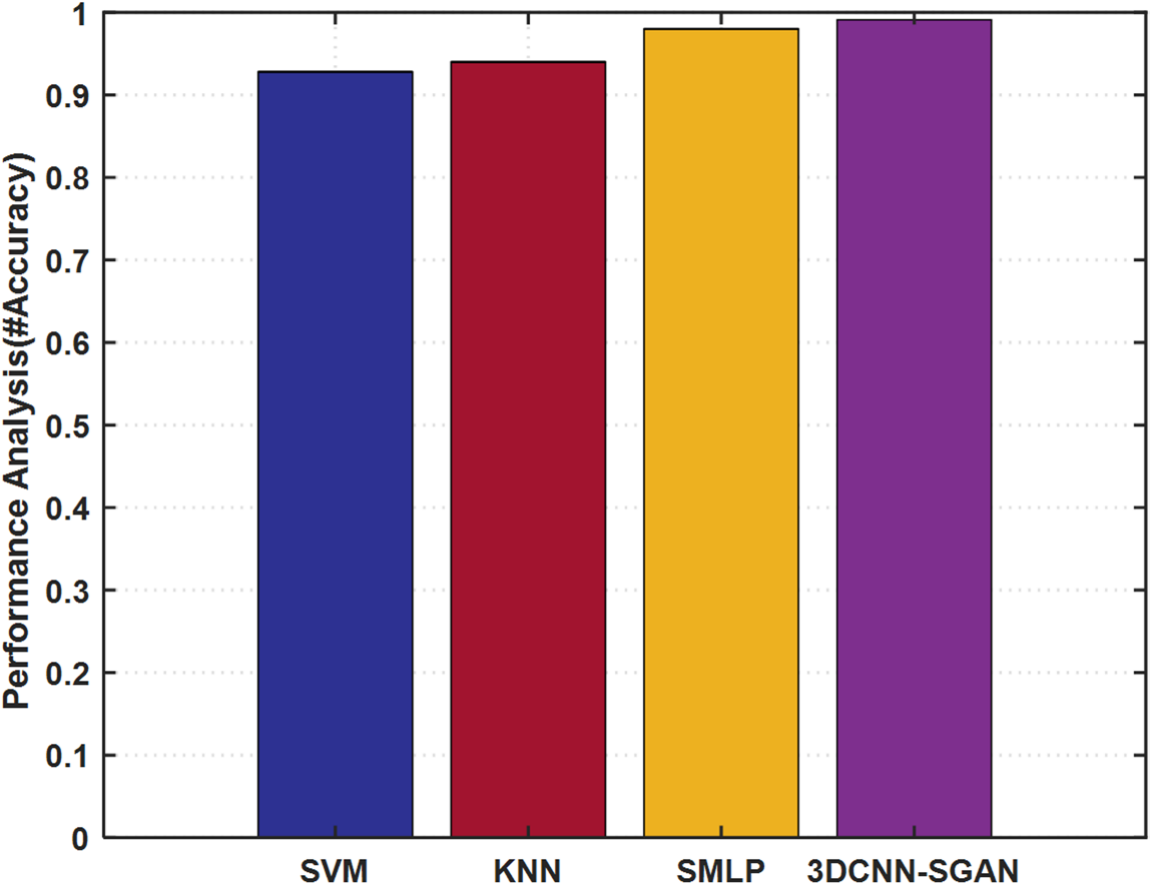
Comparison between the proposed model and traditional ML algorithms.

A comparative analysis was also done to compare the proposed 3DCNN+SGAN architecture with traditional ML, i.e., SVM, KNN, and SMLP, as shown in Fig. 9. The radial basis function (RBF) kernel was used in the SVM implementation, and the KNN model was set with *k* = 8, uniform weighting, and the use of the Euclidean distance metric. To measure the performance of a model, accuracy measures (bounded between 0 and 1) were taken, where larger values mean better classification efficacy. Despite the relatively high level of accuracy in all models, the 3DCNN+SGAN model outperformed other models, having the highest accuracy level of 99.10%.

In order to measure performance improvement, the proposed hybrid model achieved 5.63% better performance than SVM (93.80% accuracy), 7.02% better than KNN (92.60% accuracy), and 3.99% better than SMLP (95.30% accuracy) in terms of classification accuracy. Regarding regression results, using root mean square error (RMSE), the hybrid solution showed a 27% improvement over SVM, a 30% worse performance than KNN, and a 22% worse performance than SMLP. These improvements support the ability of the hybrid model to learn deep spatiotemporal features and generalize to diverse gas-mixture scenarios. By incorporating SGANs, the model effectively benefits from both labeled and synthetic data, enhancing its stability and providing greater resistance to sensor noise and variability in mixed gas concentrations.

The SMLP outperformed all other traditional models and was markedly superior to SVM and KNN. This advantage stems from its hierarchical structure, which enables more effective modeling of complex, nonlinear relationships and interdependencies among sensor readings. Despite this good performance, the SMLP did not capture enough of the spatial and temporal relationships that are most important for successful mixed-gas detection, which is also addressed by the 3DCNN+SCAN framework proposed in this paper. This is due to its 3D convoluted layers, which are more skilled at outlining spatial patterns, as well as the SCAN mechanism, which improves feature representation and thus allows for more detailed discrimination of complex datasets. As a result, the 3DCNN+SCAN architecture is particularly well suited for mixed-gas sensor analysis, highlighting the practical advantages of advanced deep learning architectures in analyzing complex sensor signals.

To evaluate the robustness of the proposed SGAN framework with respect to the underlying CNN design, we performed a sensitivity analysis by varying key architectural parameters, including the number of convolutional layers, pooling strategies, filter sizes, and dropout rates. The results shown in Table 1 indicate that classification accuracy and F1-score generally increase with the depth of the CNN up to four convolutional layers, after which performance gains saturate and overfitting becomes more likely.

**Table 1:** Effect of CNN architectural variations.

CNN variant	Accuracy (%)	F1-score	Notes
2 Conv layers, MaxPool, 3 × 3	92.1	0.91	Shallow baseline
4 Conv layers, MaxPool, 3 × 3	95.4	0.94	Best balance of depth and generalization
6 Conv layers, MaxPool, 3 × 3	95.6	0.94	Slight gain, higher training cost
4 Conv layers, AvgPool, 3 × 3	93.8	0.92	Avg pooling loses discriminative info
4 Conv layers, MaxPool, 5 × 5	94.0	0.92	Larger filters less effective
4 Conv layers, MaxPool, 7 × 7	92.5	0.91	Over-smoothing effect
4 Conv layers, MaxPool, 3 × 3 + Dropout 0.3	95.8	0.95	Best overall
4 Conv layers, MaxPool, 3 × 3 + Dropout 0.6	93.0	0.91	Underfitting due to high dropout

Empirical analysis shows that average pooling achieves only slightly lower accuracy than max pooling, implying that max pooling is more effective at preserving the discriminative characteristics of sensor response patterns. In addition, doubling the convolutional kernel to 5 × 5 did not lead to significant improvement in performance, but putting excessively huge kernels of 7 × 7 actually led to a reduction in performance, supporting the idea that local feature extraction is working more favorably in gas mixture discrimination. It was found that dropout was beneficial in reducing overfitting, where the best rates lay in the range of 0.3 to 0.5; too large dropouts (greater than 0.6) caused underfitting and a drop in performance. These results demonstrate that in spite of the fact that CNN hyperparameters alter quantitative results, the presented SGAN continues to outperform baseline supervised CNNs in all the considered architectural designs, which underscores its resilience and generalization ability. To further confirm the accuracy of the reported accuracies, we performed a paired *t*-test on the five-fold cross validation results of datasets D1 and D2. The *t*-test of test accuracies showed that the D1(0.88 + 0.01) was significantly better than D2(0.85 + 0.01) at *t*(4) = 5.72, *p* = 0.0046. Since the *p*-value obtained is significantly lower than the traditional 0.05, we can assume that the difference in performance observed is statistically significant and not the result of a random variation. These findings support the effectiveness of the proposed method and highlight the model’s robustness across different dataset partitions.

## Conclusion

This paper presented a hybrid 3DCNN-SGAN to enhance the classification and detection of gas mixture concentrations in actual atmospheric conditions. Through the integration of spatiotemporal feature extraction capabilities of 3DCNNs and SGANs, the proposed model performed well and consistently in five-fold cross-validation. On the D1 dataset, it had an average test accuracy of 0.88 ± 0.01, and on the D2 dataset, it had an average of 0.85 ± 0.01, which validates the strength and generalizability of the framework. The hybrid model had up to 7% higher accuracy and lower RMSE values than traditional baseline models (SVM, KNN, and SMLP), which proved the effectiveness of the hybrid model in the case of complex sensor data and the small number of labelled samples. There are, however, certain shortcomings. The present procedure is based on a particular sensor configuration and controlled conditions of data, and it might not be applicable to various real-life settings. Furthermore, the training of SGANs is computationally expensive and needs attention to parameters so that they do not blow up.

Although the suggested framework has strong results on controlled dynamic gas-mixture data, it does not specifically address long-term sensor drift, uncontrolled fluctuations in humidity and temperature, or cross-device generalizability. The SGAN component is mainly sensitive to the insufficiency of labels and the improved learning of representations, but it is not aimed at combating aging of physical sensors. Therefore, future research will focus on domain adaptation and drift-resistant learning approaches to extend the research to longer and more real-world applications. Also, future research should focus on extending generalization across heterogeneous sensor platforms and variable environmental conditions, potentially through domain adaptation strategies. Expanding the framework to support multi-class and multi-gas scenarios, along with integrating it into real-time IoT platforms, would further enhance its scalability and practical deployment for intelligent gas monitoring systems.

## Data Availability

Data sets generated during the current study are available from the corresponding author (Ghazala Ansari) on reasonable request.
